# Supplementary material for: Patients’ Preferences and Willingness to Pay for Solid Forms of Oral Medications—Results of the Discrete Choice Experiment in Polish Outpatients
Source: Pharmaceutics. 2020 Mar 6;12(3):236. doi: 10.3390/pharmaceutics12030236 (PMC7150858; doi:10.3390/pharmaceutics12030236)
Supplement: Supplementary file 1 [file pharmaceutics-12-00236-s001.pdf]

pharmaceutics

MDPI

Supplementary Materials: Patients' Preferences and Willingness to Pay for Solid Forms of Oral Medications—Results of the Discrete Choice Experiment in Polish Outpatients

Marta Kurczewska-Michalak, Przemysław Kardas and Mikolaj Czajkowski

Table S1. Sixteen choice tasks presented to the respondents.

CHRONIC									
A					B				
**Choice situation**	**Form**	**Size**	**Color**	**Price**	**Choice situation**	Form	Size	Color	Price
**P1**	long	small	white	5	**P1**	round	medium	red	20
**P2**	round	large	white	15	**P2**	caps	small	blue	10
**P3**	round	small	red	15	**P3**	long	large	white	10
**P4**	long	small	red	5	**P4**	caps	large	yellow	20
**P5**	caps	medium	yellow	10	**P5**	round	large	white	15
**P6**	caps	large	yellow	20	**P6**	round	medium	blue	5
**P7**	caps	medium	blue	10	**P7**	long	small	red	15
**P8**	round	large	red	20	**P8**	long	small	yellow	5
ANTIBIOTIC									
A					B				
**Choice situation**	Form	Size	Color	Price	**Choice situation**	Form	Size	Color	Price
**P1**	caps	small	white	20	**P1**	round	medium	yellow	5
**P2**	round	small	blue	10	**P2**	caps	medium	white	15
**P3**	long	medium	red	15	**P3**	round	large	blue	10
**P4**	round	medium	white	5	**P4**	long	large	blue	20
**P5**	round	small	yellow	10	**P5**	long	medium	red	15
**P6**	long	large	yellow	20	**P6**	caps	small	white	10
**P7**	caps	medium	blue	15	**P7**	round	small	yellow	5
**P8**	long	large	blue	5	**P8**	caps	small	red	20

Table S2. Socio-demographic characteristics of the study participants.

**Gender**		
**Male**	98	49.0%
**Female**	102	51.0%
**Age groups in years**		
**20 – 29**	22	11.0%
**30 – 49**	7	35.5%
**50 – 69**	92	46.0%
**70 – 84**	12	6.0%
**Missing data**	3	1.5%
**Educational level**		
**Primary**	8	4.0%
**Secondary**	113	56.5%
**University degree**	79	39.5%
**Monthly income level (PLN)**		

Less than 2000	74	37%
2000 – 2499	39	19.5%
2500 – 3499	35	17.5%
3500 – 4499	14	7%
4500 – 8999	9	4.5%
9000 – 14999	3	1.5%
More than 15000	1	0.5%
Missing data	25	12.5%
Current chronic therapy		
Yes	103	51.5%
No	97	48.5%
Occupation		
Student	6	3%
Employed	127	63.5%
Unemployed	1	0.5%
Pensioner	61	30.5%
Other	5	2.5%
Place of residence		
City over 500 inhabitants	145	72.5%
City 100 – 500 inhabitants	2	1%
City 50 – 100 000 inhabitants	15	7.5%
Town less than 50 000 inhabitants	13	6.5%
Countryside	25	12.5%
**Total**	200	100.0%

Table S3. The results of the multinomial logit model with socio-demographic interactions, representing patients WTP (in EUR per week) for various forms of short-term and chronic treatment medications.

Attributes	Short-term treatment				Chronic treatment			
	Main effect (st. err.)	Female (st. err.)	Age (st. err.)	Permanent pharm. (st. err.)	Mean (st. err.)	Female (st. err.)	Age (st. err.)	Permanent pharm. (st. err.)
Form – long tablet (vs. round tablet)	2.54** (1.02)	1.09** (0.52)	−0.20 (0.22)	0.40 (0.62)	1.57*** (0.47)	0.14 (0.29)	−0.33*** (0.11)	0.80** (0.31)
Form – capsule (vs. round tablet)	0.90 (1.16)	1.15* (0.59)	0.10 (0.25)	−0.16 (0.72)	1.93*** (0.43)	0.19 (0.28)	−0.29*** (0.09)	0.50* (0.28)
Size – medium (vs. small)	−2.12** (0.97)	−0.10 (0.54)	−0.26 (0.20)	1.05* (0.60)	−2.43*** (0.48)	−0.89** (0.34)	−0.06 (0.11)	0.65** (0.33)
Size – large (vs. small)	−4.39*** (0.75)	−0.85** (0.41)	−0.03 (0.15)	0.68 (0.46)	−6.18*** (0.73)	−1.98*** (0.60)	0.34** (0.16)	0.09 (0.48)
Color – yellow (vs. white)	1.74 (1.07)	0.50 (0.54)	0.04 (0.23)	−0.56 (0.64)	0.71 (0.70)	−0.45 (0.49)	−0.21 (0.15)	0.96** (0.45)
Color – blue (vs. white)	1.90** (0.85)	−0.07 (0.47)	−0.22 (0.17)	−0.22 (0.51)	−0.87 (0.57)	−0.42 (0.38)	−0.04 (0.12)	0.41 (0.37)
Color – red (vs. white)	2.51* (1.35)	-0.49 (0.70)	-0.34 (0.27)	0.51 (0.80)	−2.49*** (0.60)	−0.47 (0.38)	0.17 (0.11)	0.43 (0.35)
